# Proteasome augmentation is protective against Alzheimer’s disease cognitive decline

**DOI:** 10.1002/alz70861_108979

**Published:** 2025-12-23

**Authors:** Kanisa Davidson, Danitra Parker, Andrew Pickering, Maria Gaczynska, Pawel Osmulski

**Affiliations:** ^1^ the university of texas health science center at houston, Houston, TX USA; ^2^ The University of Texas Health Science Center at Houston, houston, TX USA; ^3^ University of Texas Health Houston, Houston, TX USA; ^4^ UT Health Science Center San Antonio, San Antonio, TX USA

## Abstract

**Background:**

Alzheimer’s Disease (AD) is a progressive neurodegenerative disease that affects around 50 million people. The pathological hallmarks of AD diseases include cognitive decline and the accumulation of β‐amyloid plaques and hyperphosphorylated tau. AD treatment options are limited highlighting the need for novel approaches. Proteasomal activity is decreased across human and animal models of AD. Proteasome inhibition has been shown to increase cognitive and behavior deficits. We hypothesize that β‐amyloid and/or hyperphosphorylated tau impair proteasome function in AD by disrupting the proteasome’s capacity to degrade critical synaptic proteins for memory formation and synaptic plasticity .

**Method:**

We investigated the role of Aβ in the modulation of proteasomal function, the capacity of two proteasome‐activating compounds to rescue Aβ‐induced survival deficits in cell culture, and Aβ‐induced cognitive deficits in *Drosophila* and mice.

**Result:**

We demonstrate β‐amyloid inhibits 20S proteasome function while simultaneously driving disassembly of 26S proteasome into free 20S proteasome. Treatment with proteasome activators enhances 20S and 26S proteasome function and reduces cell death caused by Aβ42 toxicity in SK‐N‐SH cells (*n* =15, *p* < 0.05, *p* < 0.01, and *p* < 0.001). In our AD *Drosophila* models, our proteasome agonist delayed mortality and restored cognitive function (*n* =89‐150, *p* < 0.05, *p* < 0.01, and *p* < 0.001). Chronic treatment with proteasome activators protected against deficits in working memory caused by Aβ42 in mice and in hAPP(J20) mice with established deficits, acute drug treatment significantly improved spatial learning deficits, with treated mice performing comparably to controls (*n* =9‐11, *p* < 0.05).

**Conclusion:**

Our proteasome activator shows promise in mitigating AD‐like deficits by protecting against amyloid toxicity and enhancing proteasome function. These findings suggest that targeting proteasome activity could be a viable therapeutic approach for AD, warranting further investigation into the broader impacts of proteasome modulation on AD pathology.

